# Effects of estradiol on the virulence traits of *Porphyromonas gingivalis*

**DOI:** 10.1038/s41598-022-17019-z

**Published:** 2022-08-16

**Authors:** Kartheyaene Jayaprakash Demirel, Alessandra Neves Guimaraes, Isak Demirel

**Affiliations:** 1 Department of Odontological Research, Public Dental Service, Faculty of Medicine and Health, Örebro University, Campus USÖ, 701 82 Örebro, Sweden; 2 Department of Periodontology and Implantology, Public Dental Service, Faculty of Medicine and Health, Örebro University, Örebro, Sweden; 3 iRiSC-Inflammatory Response and Infection Susceptibility Centre, Faculty of Medicine and Health, Örebro University, Örebro, Sweden; 4 School of Medical Sciences, Örebro University, Örebro, Sweden

**Keywords:** Microbiology, Infectious diseases

## Abstract

*Porphyromonas gingivalis* has been strongly associated to active periodontitis sites. A number of studies have tried to elucidate the association between female steroid sex hormones and gingival health. However, until now, there is limited knowledge on estradiol effects on the virulence traits of *P. gingivalis*. The aim of the study was to investigate the impact of estradiol exposure on the virulence characteristics of *P. gingivalis* strain W50. We found that a pre- and postmenopausal concentration of estradiol increased the growth and biofilm formation of *P. gingivalis* W50. We also found that estradiol increased the release of lysine and arginine gingipains from W50. We then showed that IL-1β, CXCL10 and TGF-β1 release from gingival epithelial cells was significantly lowered by W50 pre-exposed to estradiol compared to W50 alone. Real time-qPCR showed that the gene expression of IL-18, IL-6, IL-8, TGF-β1 and NLRP3 in gingival epithelial cells was significantly lowered by W50 pre-exposed to estradiol compared to W50 alone. We also found that estradiol in a dose-dependent manner increased *P. gingivalis* colonization and invasion of gingival epithelial cells. Taken together, our findings show that estradiol has the ability to alter the virulence traits of *P. gingivalis*.

## Introduction

Oral cavity is a complex ecosystem comprising of more than 700 species of bacteria, of which *Porphyromonas gingivalis* is an established member^1,2^. The red complex species include *P. gingivalis, Treponema denticola *and* Tannerella forsythia* have been strongly tied to progression of periodontitis^3,4^. Several studies have also tried to investigate the association between female steroid sex hormones and gingival health^5,6^. Periodontitis has a complex etiology and much remains to be elucidated. Globally, there has been a demonstrative increase in life expectancy, and this alters the demographics of females in their postmenopausal stage^7^. Geriatric follow-up studies have established that periodontitis in early old age tends to be associated to an increase in general mortality in older age^8^.

Estrogen has vital functions, and its levels are fluctuating during childbearing age. Higher estrogen levels during pregnancy promote gingivitis and low postmenopausal levels predispose women to increased alveolar bone loss^9^. The human gingival fibroblasts/epithelial cells and periodontal ligamental cells express ER-β (Estrogen-beta subtype) receptors^10^. It has been speculated that normal serum levels of estrogen are important for the maintenance of healthy periodontium and the absence of which could be correlated to periodontal disease. Some of the estrogen-mediated effects on the periodontium are; increased vascularisation, stimulation of osteoblasts, suppression of neutrophil functions and T-cell responses, and active modulation of pro-inflammatory cytokines^11–14^. Of estrone, estradiol and estriol, estradiol is the most potent and abundant estrogen in women of reproductive age. Serum estradiol levels range between 30–400 pg/ml in premenopausal women and falls below 20 pg/ml in postmenopausal women. The reference range for saliva estradiol is 3–12 pg/ml in premenopausal women and 0.5–1.7 pg/ml in postmenopausal women^15,16^.

*P. gingivalis* is an established marker of periodontal disease in old age^1,4^. Periodontitis is a polymicrobial infection and its associated systemic co-morbidities have been well documented^17^. Among the several virulent traits, *P. gingivalis* lipopolysaccharide, fimbriae, gingipains, peptidylarginine deiminase, serine phosphatase, nucleoside diphosphate kinase and hemagglutinins are the most recognized factors. These factors contribute to adhesion, invasion and modification of host defence mechanisms^18^. The focus of majority of research conducted today in the field of host–pathogen interaction is on elucidating how pathogens, with their respective virulence factors, successfully modulate or evade the immune responses to cause infections. However, less is known about how host immune factors and hormones are affecting the virulence of *P. gingivalis* by cross-kingdom interactions.

The majority of the current research conducted on periodontal disease and estrogen, have focused on the effects on the human host and evaluation of risk factors. The effect of host mediators, like hormones and cytokines, on bacteria is a relatively unexplored field. It has been shown that human cytokines can bind to *Neisseria meningitides* DNA and alter its gene expression^19^. We have previously shown that pro-inflammatory cytokines and estradiol alters uropathogenic *E. coli* virulence^20,21^. Estrogen has been associated with increased growth and survival of several Gram-negative bacteria^20,21^. *Pseudomonas aeruginosa* (*P. aeruginosa*) exposure to estradiol resulted in a virulent mucoid biofilm phenotype. This shift was due to a constitutive cytosolic estrogen-binding protein expressed by *P. aeruginosa*^22^. *Chlamydia trachomatis* exposure to estradiol resulted in a downregulation of genes associated with nucleotide metabolism and fatty acid biosynthesis, and upregulation of genes linked to stress responses^23^. We know that there is a clinical association between estradiol levels and the development of periodontal disease^5,6^. However, how the direct effects of estradiol on *P. gingivalis* affects the development of periodontal disease is unknown.

Understanding how the human host affects the virulence of *P. gingivalis* may be a new frontier in the fight against *P. gingivalis* associated infections. If we can unravel how *P. gingivalis* senses its microenvironment and activates its virulence, we may be able to prevent or dampen this activation, which in turn could prevent the progression of the infection. However, until now, there is limited knowledge of how estradiol affects the virulence of *P. gingivalis*. The aim of the study was to investigate the impact of estradiol exposure on the virulence of *P. gingivalis*.

## Materials and methods

### Bacterial and cell culture

*P. gingivalis* strain W50 kindly provided by Professor M. A. Curtis, (Molecular Pathogenesis Group, Queen Mary, University of London), was used in this study. *P. gingivalis* W50 was anaerobically cultured at 85% N_2_, 5% CO_2_, and 10% H_2_ at 37 °C in an anaerobic chamber (Concept 400-M Anaerobic Workstation; Ruskinn Technology Ltd., Leeds, UK) in tryptic soy broth (TSB) supplemented with yeast extract (1 mg/ml), hemin (5 μg/ml) and menadione (1 μg/ml). *P. gingivalis* W50 was grown for 48 h and then centrifuged for 10 min at 10,000 rpm, washed and resuspended with PBS.

The human gingival epithelial cell (GEC) line Ca9-22 (Tebu-bio, Paris, France) was grown in Dulbecco’s Modified Eagle Medium (DMEM) (Lonza, Basel, Switzerland) with 10% fetal bovine serum (FBS), 2 mM l-glutamine, 1 mM non-essential amino acids (Thermo Fisher Scientific, Waltham, MA, USA) and incubated at 37 °C and 5% CO_2_ atmosphere. The culture medium was changed during experiments to DMEM with 2% FBS, 1 mM non-essential amino acids and 2 mM l-glutamine, as previously described^2,24^.

### Bacterial growth assessment

*P. gingivalis* W50 (1 × 10^6^ CFU/ml) was grown in TSB supplemented with yeast extract (1 mg/ml), hemin (5 μg/ml) and menadione (1 μg/ml) with or without the presence of 17β-Estradiol [1 pg/ml, 2 pg/ml, 5 pg/ml, 10 pg/ml, 100 pg/ml, 300 pg/ml and 1000 pg/ml (E2758, Sigma-Aldrich, St. Louis, MO, USA)] in a 96-well plate. The 96-well plate was then incubated at 37 °C anaerobically and the optical density (600 nm) was measured using a spectrophotometer (Cytation 3, Biotek Inc, Winooski, VT, USA), as previously described^20,21^.

### Biofilm and gingipain measurement

After the growth assessment, the same 96-well plate was used for investigating biofilm formation. The plate was washed three times with sterile RO-water and 0.1% Crystal violet (Thermo Fisher Scientific) was then added to the wells for 20 min in order to stain the biofilm. The plate was then washed with RO-water and left to dry overnight. Ethanol (95%) was used to dissolve the crystal violet and the absorbance (540 nm) was measured by a spectrophotometer (Cytation 3).

After assessing the bacterial growth, the TSB was collected from the same 96-well plate and centrifuged at 10,000 rpm for 10 min in order to measure gingipain activity. The bacterial-free TSB was then transferred into a new 96-well plate and 100 µM Z-His-Glu-Lys-AMC (substrate for Lysine-Gingipain, PeptaNova GmbH, Germany) or Boc-Phe-Ser-Arg-AMC (substrate for Arginine-Gingipain, PeptaNova GmbH) was added to the plate. The plate was incubated for 1 h at 37 °C and the gingipain activity was measured at 340/440 nm (Cytation 3).

### Cytokine release and viability assay

*P. gingivalis* W50 was grown in the presence or absence of estradiol for 48 h at 37 °C as previously stated. Estradiol was then washed way from *P. gingivalis* W50 with PBS and the gingival epithelial cell line was infected with the respective treatment at Multiplicity of infection (MOI) of 100 for 24 h at 37 °C and 5% CO_2_. Supernatants were collected after the infection and centrifuged for 10 min at 10,000 rpm and stored at − 80 °C. An enzyme-linked immunosorbent assay (ELISA) was performed to measure Interleukin-1β (IL-1β), CXCL10 and TGF-β1 release from the GECs. The cytokine was measured with the IL-1β and CXCL10 kits (ELISA MAX Deluxe Sets, BioLegend, San Diego, CA, USA) and TGF-β1 kit (Duo set, ELISA, R&D Systems, Minneapolis, MN, USA) according to the kit’s instructions. The concentrations were determined by optical density at 450 nm. Cell viability after *P. gingivalis* W50 infection was assessed by optical density at 490 nm using the Pierce LDH cytotoxicity assay (Thermo Fisher Scientific) according to the kit’s instructions, as previously described^20,25^.

### RNA isolation, cDNA generation and quantitative polymerase chain reaction

*P. gingivalis* W50 was grown in the presence or absence of estradiol for 48 h at 37 °C as previously stated. Estradiol was then washed way from *P. gingivalis* W50 with PBS and the gingival epithelial cell line was infected with the respective treatment at MOI of 100 for 24 h at 37 °C and 5% CO_2_. Total RNA was isolated using the E.Z.N.A^®^ Total RNA Kit I (Omega Bio-tek, Inc., Norcross, GA, USA) according to manufacturer’s instructions. RNA purity and concentration were measured using a spectrophotometer (Nano Drop 2000, Wilmington, NC, USA). 100 ng total RNA was used for the cDNA synthesis (High-Capacity cDNA Reverse Transcription Kit, Applied Biosystems, CA, USA). For the RT-qPCR, 5 ng cDNA and 250 nM of primer (Table 1) (Eurofins MWG Synthesis GmbH, Ebersberg, Munich, Germany) was used with Maxima SYBR Green qPCR Master Mix (ThermoFisher Scientific). Amplification was done using the following protocol for 40 cycles: Denaturation at 95 °C for 15 s, annealing at 60 °C for 30 s and extension at 72 °C for 30 s. A CFX96 Touch™Real-Time PCR Detection System (Biorad, CA, USA) was used. A dissociation curve between 60 and 95 °C was also done after the qPCR. CT-values were obtained and the ΔΔCt method (2^−ΔΔCt^) was used to calculate the fold difference. The results were normalized to the endogenous control GAPDH, as previously described^20,25^.

**Table 1:** Primers used for quantitative real-time PCR.

Gene symbol	Oligonucleotide sequences (5′–3′)
*pro-IL-1β*	*F:* CCACAGACCTTCCAGGAGAATG *R:* GTGCAGTTCAGTGATCGTACAGG
*pro-IL-1α*	*F:* TGTATGTGACTGCCCAAGATGAAG *R:* AGAGGAGGTTGGTCTCACTACC
*IL-18*	*F:* GATAGCCAGCCTAGAGGTATGG *R:* CCTTGATGTTATCAGGAGGATTCA
*IL-6*	*F:* AGACAGCCACTCACCTCTTCAG *R:* TTCTGCCAGTGCCTCTTTGCTG
*IL-8*	*F:* GAGAGTGATTGAGAGTGGACCAC *R:* CACAACCCTCTGCACCCAGTTT
*TGF-β1*	*F:* TACCTGAACCCGTGTTGCTCTC *R:* GTTGCTGAGGTATCGCCAGGAA
*NLRP3*	*F:* GGACTGAAGCACCTGTTGTGCA *R:* TCCTGAGTCTCCCAAGGCATTC
*GAPDH*	*F:* GTCTCCTCTGACTTCAACAGCG *R:* ACCACCCTGTTGCTGTAGCCAA

### Colonization assay

*P. gingivalis* W50 was grown anaerobically in the presence or absence of estradiol (1 pg/ml and 100 pg/ml) for 48 h at 37 °C. Estradiol was then washed way from *P. gingivalis* W50 with PBS. The bacteria were then FITC-labeled (Sigma-Aldrich) and the GECs were infected with the respective treatment at MOI of 100 for 2 h at 37 °C and 5% CO_2_ to measure bacterial colonization (adherent and intracellular bacteria). After which the cells were washed with PBS and the FITC-labeled *P. gingivalis* W50 were quantified with the Cytation 3 plate reader. Colonization is presented as % mean fluorescence intensity (MFI) of *P. gingivalis* W50, as previously described^20^.

### Invasion assay

*P. gingivalis* W50 was grown anaerobically in the presence or absence of estradiol (5 pg/ml and 100 pg/ml) for 48 h at 37 °C. Estradiol was then washed way from *P. gingivalis* W50 with PBS and the GECs were infected with the respective treatment at MOI of 100 for 2 h at 37 °C and 5% CO_2_. After which the cells were washed with PBS. DMEM supplemented with 2% FBS, 300 μg/ml gentamicin and 200 μg/ml metronidazole were then added to the GEC to kill remaining extracellular *P. gingivalis* W50 during 1 h. The plate was then washed again, and the cells were lysed with 0.1% Triton-X 100 in PBS (with calcium chloride 100 mg/l and magnesium chloride 100 mg/l) for 10 min under gentle rotation, as previously described with minor modifications^26^. Finally, *P. gingivalis* W50 were plated on agar plates [Tryptic soy broth supplemented with agar (15 mg/ml), yeast extract (1 mg/ml), hemin (5 μg/ml) and menadione (1 μg/ml)], incubated for 48 h anaerobically at 37 °C, and the colonies were then counted.

### Statistical methods

All data shown are expressed as mean ± SEM. The differences between the groups were analyzed by student’s unpaired *t* test or one‐way ANOVA followed by Bonferroni multiple testing correction. Results were considered statistically significant at p < 0.05. n = number of independent biological experiments, as previously described^20^.

## Results

### Estradiol induces growth and biofilm formation in *P. gingivalis*

**Figure 1:**
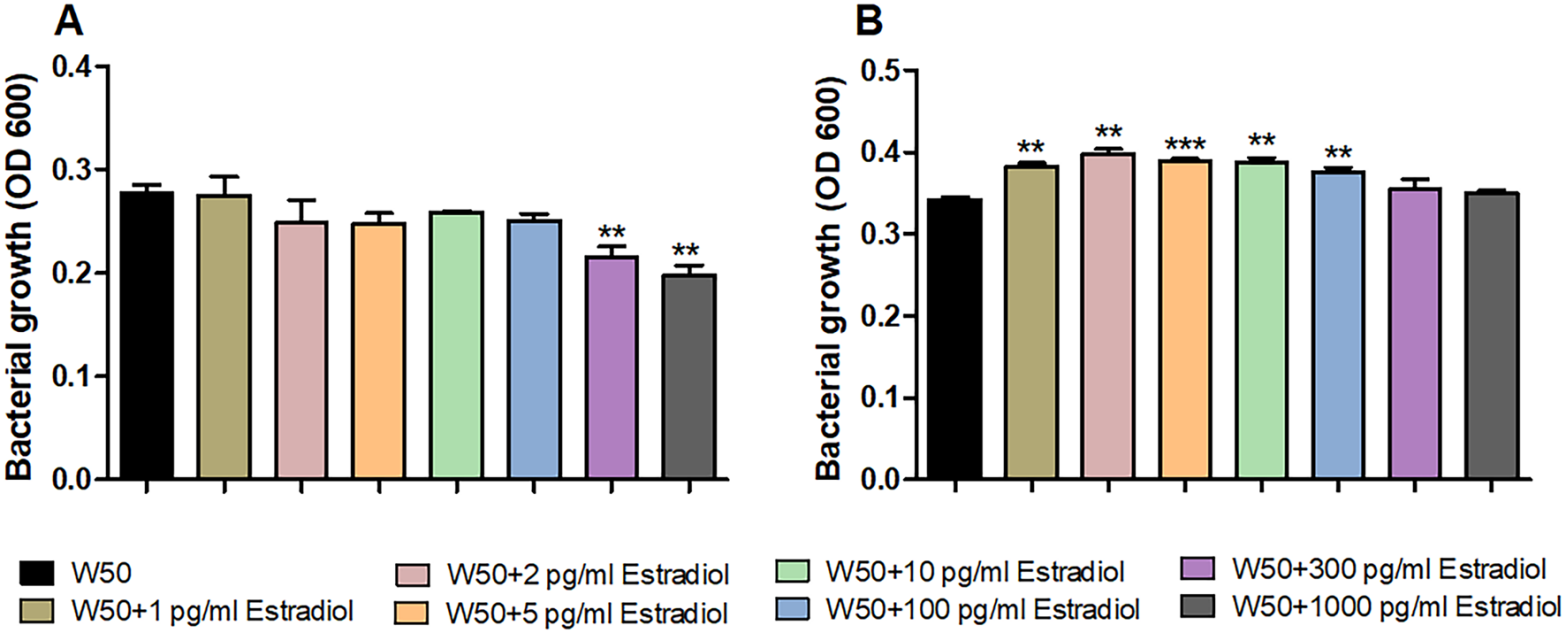
Estradiol alters *P. gingivalis* growth. *P. gingivalis* W50 growth with or without the presence of estradiol (1 pg/ml, 2 pg/ml, 5 pg/ml, 10 pg/ml, 100 pg/ml, 300 pg/ml and 1000 pg/ml,) after 24 h (**A**) and 72 h (**B**). Data are presented as mean ± SEM of n = 3 independent experiments. The asterisk distinguishes statistical significance: **p < 0.01, ***p < 0.001 vs. unstimulated *P. gingivalis* W50.

We started with evaluating the effects estradiol had on the growth of *P. gingivalis* W50. We found that 1, 2, 5, 10 and 100 pg/ml of estradiol significantly increased the growth of W50 compared to unstimulated W50 after 72 h (Fig. 1B). The higher concentrations of estradiol, 300 and 1000 pg/ml, did not induce a significant growth increase compared to unstimulated W50 after 72 h (Fig. 1B). However, we did observe a reduced growth of W50 in the presence of 300 and 1000 pg/ml estradiol compared to unstimulated *P. gingivalis* W50 after 24 h (Fig. 1A).

**Figure 2:**
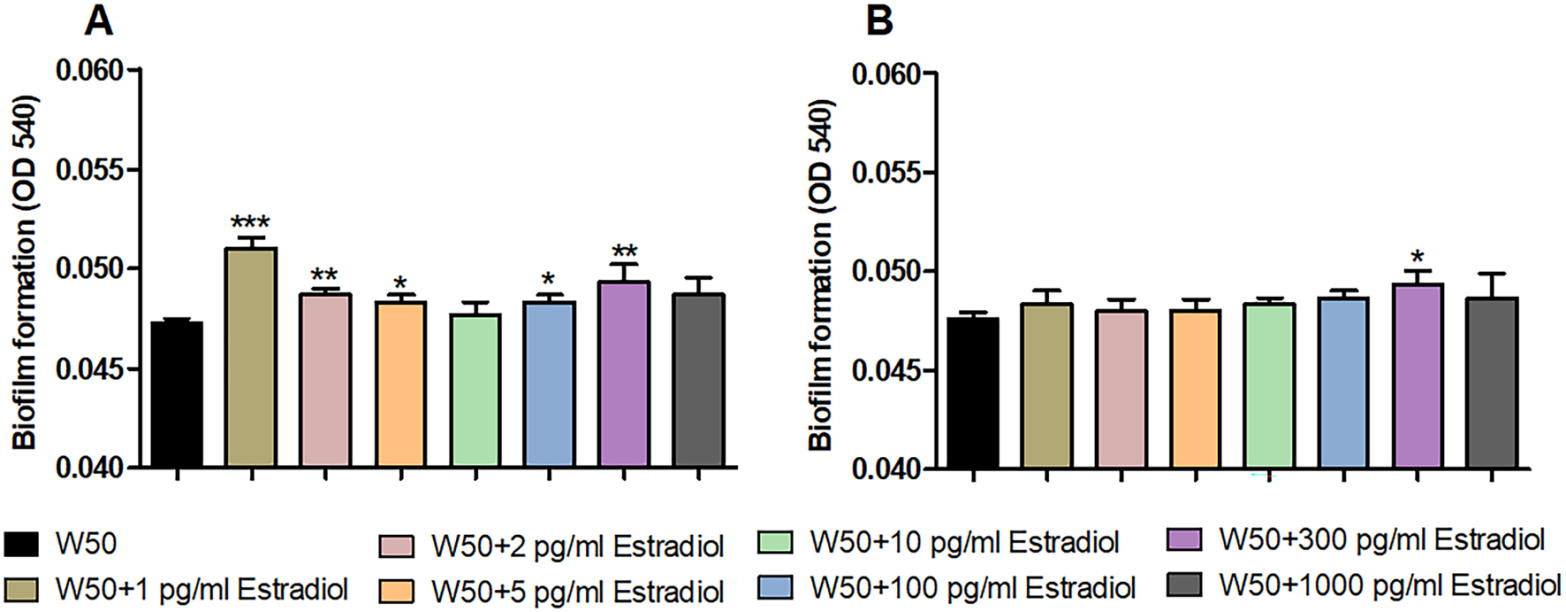
Estradiol alters *P. gingivalis* biofilm formation. *P. gingivalis* W50 biofilm formation with or without the presence of estradiol (1 pg/ml, 2 pg/ml, 5 pg/ml, 10 pg/ml, 100 pg/ml, 300 pg/ml and 1000 npg/ml,) after 24 h (**A**) and 72 h (**B**). Biofilm was measured using crystal violet absorption method. Data are presented as mean absorbance at 540 nm, mean ± SEM of n = 3 independent experiments. The asterisk distinguishes statistical significance: *p < 0.05, **p < 0.01, ***p < 0.001 vs. unstimulated *P. gingivalis* W50.

We proceeded by investigating the effects of estradiol on *P. gingivalis* biofilm formation. The biofilm formation was significantly increased by 1, 2, 5, 100, 300 pg/ml after 24 h (Fig. 2A) and 300 pg/ml estradiol after 72 h (Fig. 2B) compared to unstimulated W50. The biggest biofilm difference was observed with 1 pg/ml estradiol compared to unstimulated *P. gingivalis* W50 after 24 h (Fig. 2A).

### Estradiol augments gingipain production

**Figure 3:**
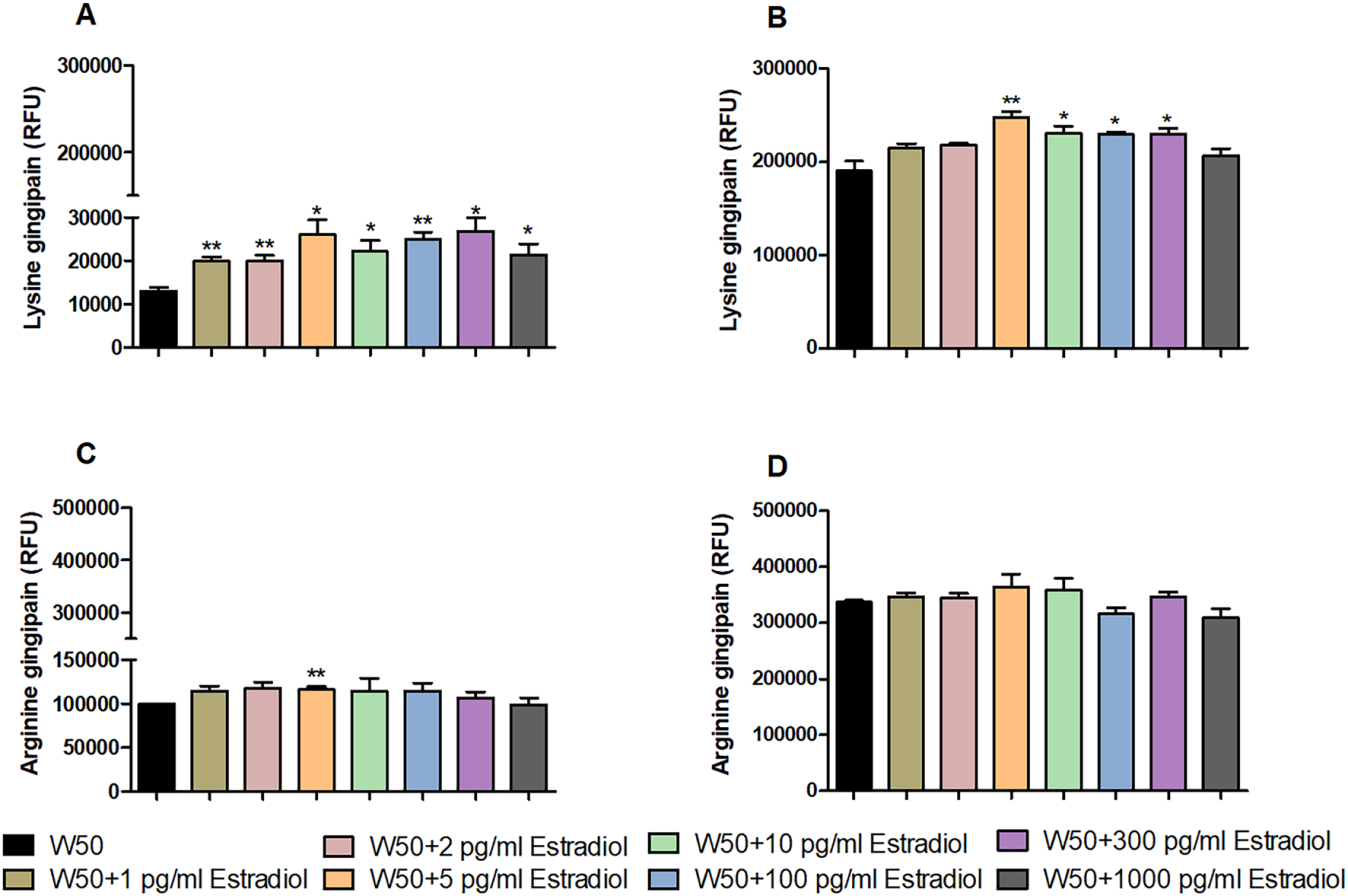
Estradiol increases the release of gingipains from *P. gingivalis*. *P. gingivalis* W50 lysine and arginine gingipain activity assay with or without the presence of estradiol (1 pg/ml, 2 pg/ml, 5 pg/ml, 10 pg/ml, 100 pg/ml, 300 pg/ml and 1000 pg/ml,) during 24 h (**A**,**C**) and 72 h (**B**,**D**). Gingipain activity is measured using lysine and arginine gingipains substrate assay. Data are derived as relative fluorescence units (RFU) at 340/440 nm and presented as mean ± SEM of n = 3 independent experiments. The asterisk distinguishes statistical significance: *p < 0.05, **p < 0.01 vs. unstimulated *P. gingivalis* W50.

We proceeded with evaluating the effects of estradiol on gingipain production. After 24 h, lysine gingipain production (Fig. 3A) was increased significantly by all estradiol concentrations, and arginine gingipain production (Fig. 3C) was increased significantly by 5 pg/ml estradiol in comparison to unstimulated *P. gingivalis* W50. Whereas after 72 h, 5–300 pg/ml estradiol induce a higher lysine gingipain production comparison to unstimulated *P. gingivalis* W50 (Fig. 3B). However, no altered arginine gingipain production was observed after 72 h (Fig. 3D).

### Estradiol altered cytokine and LDH release from gingival epithelial cells

**Figure 4:**
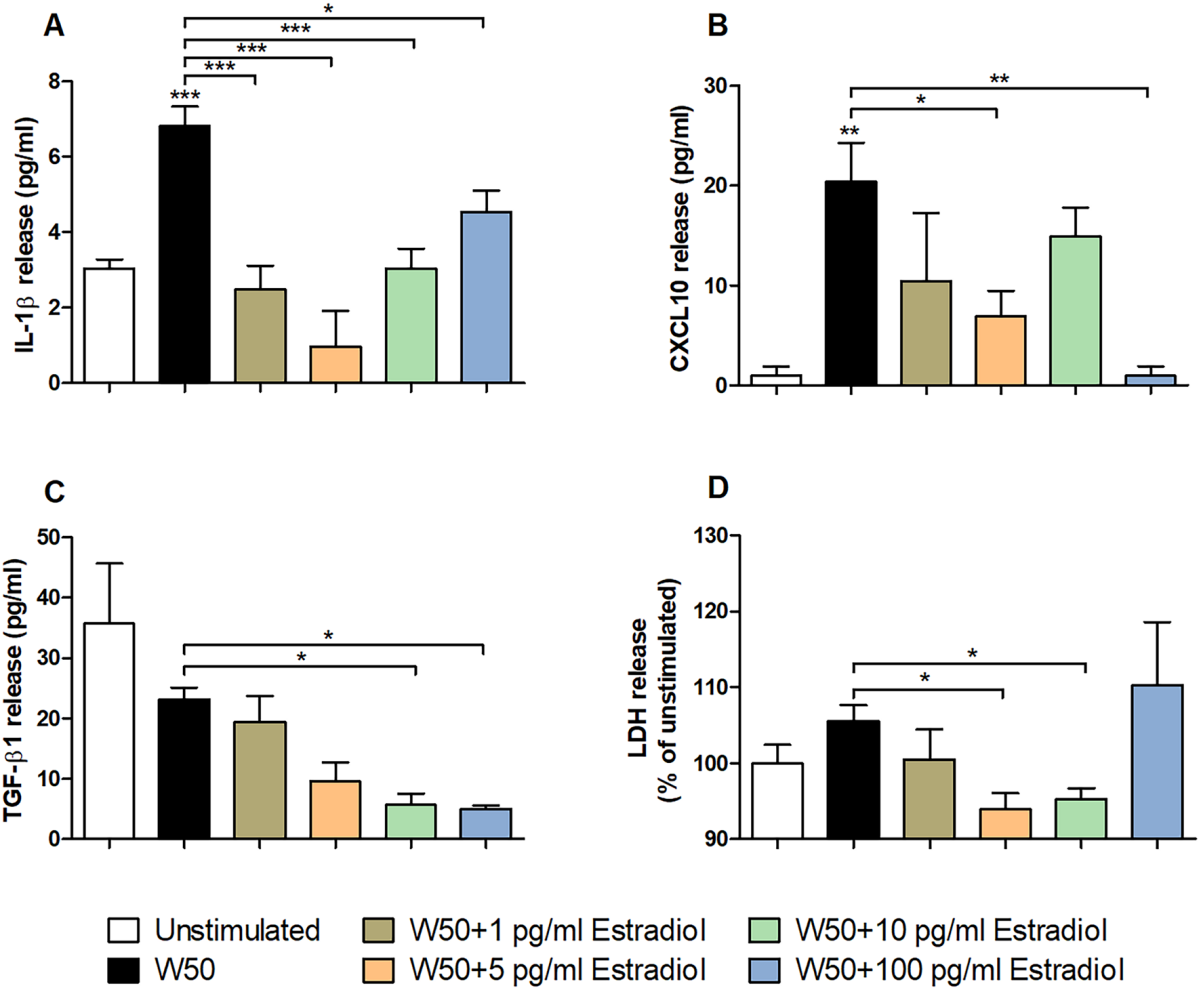
Estradiol alters *P. gingivalis* induced cytokine release. IL-1β (**A**), CXCL10 (**B**), TGFβ-1 (**C**) and LDH (**D**) release from gingival epithelial cells infected with *P. gingivalis* with or without estradiol (1, 5, 10 and 100 pg/ml) pre-treatment, after 24 h. Data are presented as mean ± SEM of n = 3 independent experiments. The asterisk distinguishes statistical significance: *p < 0.05, **p < 0.01, ***p < 0.001 vs. unstimulated gingival epithelial cells.

We continued with evaluating if estradiol stimulated *P. gingivalis* could alter the release of IL-1β, CXCL10 and TGF-β1 from gingival epithelial cells. We found that *P. gingivalis* W50 could induce a significant increased release IL-1β, CXCL10, but not TGF-β1, from gingival epithelial cells compared to unstimulated cells (Fig. 4A–C). We then found that the cytokine release was significantly lowered by *P. gingivalis* W50 stimulated with 1 pg/ml estradiol for IL-1β, 5 pg/ml estradiol for IL-1β and CXCL10, 10 pg/ml estradiol for IL-1β and TGF-β1 and 100 pg/ml estradiol for IL-1β, CXCL10 and TGF-β1 compared to unstimulated *P. gingivalis* W50 (Fig. 4A–C). Furthermore, we also found that *P. gingivalis* W50 stimulated with 5 and 10 pg/ml estradiol induced less cell damage to gingival epithelial cells compared to unstimulated *P. gingivalis* W50 (Fig. 4D).

### Estradiol alters *P. gingivalis* induced expression of genes encoding cytokines and inflammasome associated proteins

**Figure 5:**
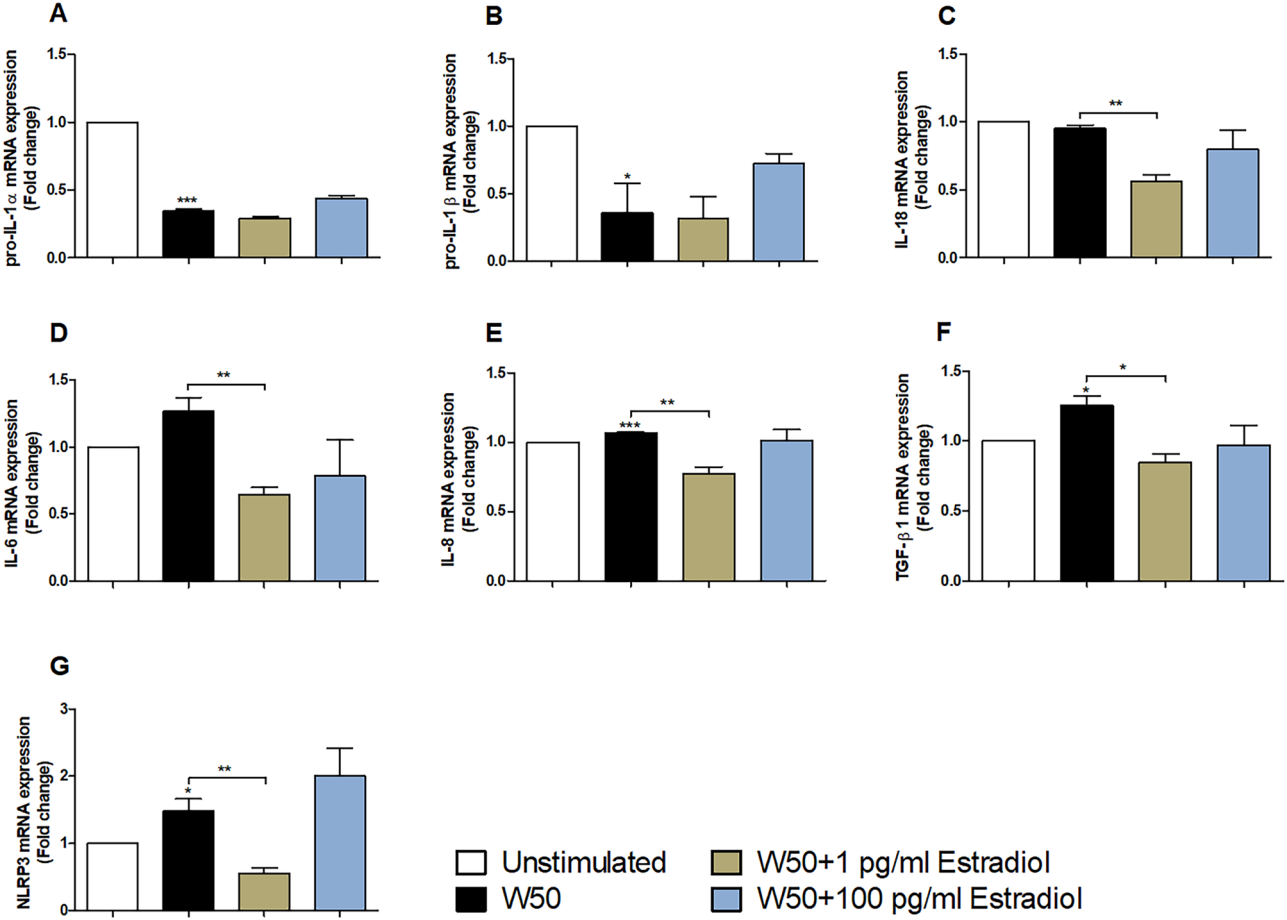
Estradiol alters *P. gingivalis* induced cytokine expression. Real-time qPCR analysis of pro-IL-1α (**A**), pro-IL-1β (**B**), IL-18 (**C**), IL-6 (**D**), IL-8 (**E**), TGFβ-1 (**F**) and NLRP3 (**G**) gene expression in gingival epithelial cells infected with *P. gingivalis* with or without estradiol (1 and 100 pg/ml) pre-treatment, after 24 h. Data are presented as mean ± SEM of n = 3 independent experiments. The asterisk distinguishes statistical significance: *p < 0.05, **p < 0.01, ***p < 0.001 vs. unstimulated gingival epithelial cells.

We found that pro-IL-1α and pro-IL-1β gene levels were significantly lowered in gingival epithelial cells in response to *P. gingivalis* W50 infection in comparison to unstimulated cells (Fig. 5A,B). We also showed that the gene expression of IL-8, TGF-β1 and NLRP3 was significantly increased in gingival epithelial cells in response to *P. gingivalis* W50 infection comparison to unstimulated cells. (Fig. 5E–G). Furthermore, we also found that the gene expression of IL-18 (Fig. 5C), IL-6 (Fig. 4D), IL-8 (Fig. 4E), TGF-β1 (Fig. 4F) and NLRP3 (Fig. 5G) in gingival epithelial cells was significantly lower in *P. gingivalis* W50 stimulated with 1 pg/ml estradiol compared to unstimulated *P. gingivalis* W50 (Fig. 5C–G). However, no significant differences in gene expression could be observed between W50 stimulated with 100 pg/ml estradiol compared to unstimulated *P. gingivalis* W50 (Fig. 5A–G).

### Estradiol increases colonization and invasion of gingival epithelial cells

**Figure 6:**
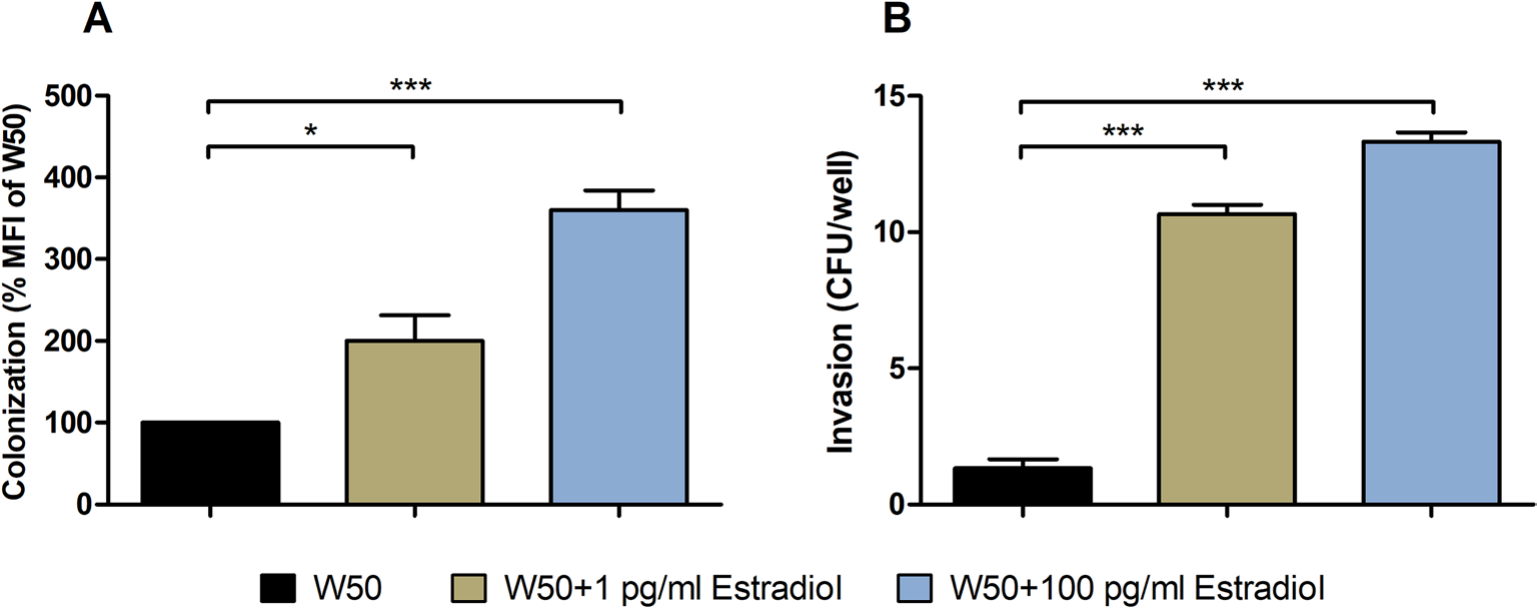
Estradiol mediated increased colonization and invasion. *P. gingivalis* W50 colonization (**A**) or invasion (**B**) of gingival epithelial cells infected with *P. gingivalis* with or without estradiol (1 and 100 pg/ml) pre-treatment after 2 h. Colonization quantification of FITC-labeled *P. gingivalis* W50 is presented as percentage mean fluorescence intensity (MFI) of W50. Invasion of gingival epithelial cells by *P. gingivalis* presents as CFU/well (colony forming units). Data are presented as mean ± SEM of n = 3 independent experiments. The asterisk distinguishes statistical significance: *p < 0.05, ***p < 0.001 vs. unstimulated *P. gingivalis* W50.

We proceeded with evaluating the effects of estradiol on *P. gingivalis* ability to colonize (adhere and invade) and invade gingival epithelial cells. *P. gingivalis* colonization seems to increase with estradiol stimulated *P. gingivalis* W50 in a dose–response manner. Both 1 and 100 pg/ml estradiol mediated a significantly increased colonization compared to unstimulated *P. gingivalis* W50 (Fig. 6A). Furthermore, we also found that estradiol increased the invasion capability of *P. gingivalis* W50 in a dose-dependent manner. Both 1 and 100 pg/ml estradiol mediated a significantly increased invasion of gingival epithelial cells compared to unstimulated *P. gingivalis* W50 (Fig. 6B).

## Discussion

A profound knowledge of host–pathogen interaction in periodontal pathology is pivotal in understanding the disease and hence, vital in developing new treatment strategies. In this study, we focus on analyzing the effects of estradiol on the virulence of *P. gingivalis* strain W50. Today we know that human factors (e.g., cytokines and hormones) produced and released by our human cells can trigger the virulence of bacteria via bacterial sensors^19^. These sensors have a dual function: host environment recognition and to initiate bacterial adaptation. It is now clear that bacterial virulence can be regulated by recognition of host factors released into the micro-environment like cytokines and hormones^19,21,27^. We know today that estrogen is important in mucosal immune responses against bacterial infections and low postmenopausal concentrations of estrogen, have been associated with alveolar bone loss in women^9^. Hence, this cross-kingdom interaction study enables us to understand the effect of host micromilieu on the bacterial virulence, which could be a new treatment frontier.

Premenopausal salivary estradiol was measured throughout the 28-day cycle and the concentrations varied between 3–12 pg/ml. Postmenopausal salivary estradiol levels were between 0.5 to 1.7 pg/ml^15,16^. Our findings showed an increase in *P. gingivalis* growth and biofilm formation by both pre- and postmenopausal concentrations of estradiol. We observed increased biofilm production after 24 h without a correlating bacterial growth. However, after 72 h, the bacterial growth was increased without increased biofilm formation. It might be that estradiol initially stimulates *P. gingivalis* to form more biofilm and later mediates a switch into a more proliferative phenotype. However, more investigation is needed to understand how estradiol affect the interplay between bacterial growth and biofilm formation. Several studies have shown a positive correlation of *P. gingivalis* prevalence to increase in female sex hormones such as estradiol and progesterone^28,29^. However, prokaryotic receptors, such as receptors on *P. gingivalis* that responds to female hormones remains to be elucidated. Previous studies have shown that estradiol can increase the growth of several gram-negative bacteria^19,20,30^ and biofilm formation^20^. Survival, growth and biofilm formation of bacteria are of clinical relevance, as biofilms allow pathogens to subvert innate immune defenses. Another important hallmark of chronic biofilm-based infections is their extreme tolerance to many conventional antimicrobial agents, making the infections more persistent^31,32^.

Gingipains are trypsin-like cysteine proteases, an essential virulence factor associated with *P. gingivalis*. Gingipains play a cardinal role in adherence, colonization, nutrient acquisition, evading host defenses and inflammatory response, tissue destruction, invasion and systemic dissemination. *P. gingivalis* strain W50 expresses two types of gingipains- (i) arginine specific and (ii) lysine specific^33^. In our study, we observed an augmentation in primarily lysine, but also arginine gingipain expression by pre- and postmenopausal concentrations of estradiol, during early and late stages of growth. However, it is interesting that after 24 h, the estradiol mediated increased gingipain release from *P. gingivalis* was not associated with increased bacterial growth. It might be that estradiol induces an active release of gingipains independent of bacterial growth. We also elucidated the augmentative effects of estradiol on *P. gingivalis* colonization and invasion of gingival epithelial cells. We found a dose-dependent increase of *P. gingivalis* colonization and invasion of gingival epithelial cells mediated by estradiol. Fimbrial, non-fimbrial adhesions and gingipains, either directly or indirectly, are involved in adhesion of *P. gingivalis* to gingival epithelial cells. The initial step of *P. gingivalis* attachment to the oral tissue is fimbriae-mediated and arginine gingipains are crucial for fimbrial maturation. In addition, gingipains mediate a tight adherence to epithelial cells and several extracellular matrix proteins, which makes them potent non-fimbrial adhesins^33,34^. Pathirana and colleagues elucidated the important function of lysine gingipains in adherence to oral epithelial cells, by using isogenic mutants of *P. gingivalis* W50 lacking the lysine gingipain. This mutant showed significantly decreased adherence to oral epithelial cells in comparison to the wild- type *P. gingivalis* W50 or its arginine gingipain mutants^34^. Taken together, estradiol promotes *P. gingivalis* colonization of gingival epithelial cells, which may help the bacteria evade the host immune responses. Thereby establish a persistent, low-grade infection and associated inflammation.

We continued to investigate if *P. gingivalis* exposed to estrogen could alter the release of cytokine from gingival epithelial cells. We found that *P. gingivalis* could induce an increased release of IL-1β and CXCL10, and that this release was lowered by *P. gingivalis* stimulated with estradiol. However, TGF-β1 was not induced by *P. gingivalis*, but we observed a dose-dependent decrease in TGF-β1 release from *P. gingivalis* stimulated with estradiol. IL-1β is a tightly regulated and potent pro-inflammatory cytokine. Pro-IL-1β requires maturation mediated by the inflammasome prior to its release^35^. Infectious pathogens or toxic chemicals that result in inflammation, activate innate immune signaling through direct or indirect activation of pattern recognition receptors such as toll-like receptors and NOD-like receptors (NLRs)^36^. NLRP3 is a NOD-like receptor that can interact with apoptosis-associated speck-like protein containing a caspase-recruitment domain (ASC) and caspase-1 resulting in the assembly of an inflammasome complex, which is up-stream of IL-1β maturation and release^37,38^. CXCL10 is significantly increased in periodontitis subjects and CXCL10 has been proposed as a marker for periodontal disease^39^. In patients with periodontitis, a significantly higher concentration of TGF-β1 was present in the gingival tissues and fluid samples obtained from deeper periodontal pockets than in the less involved sites. TGF-β1 is present in gingivitis and even detected during the early onset of periodontitis. TGF-β1 plays a vital role in wound healing and immune responses^40^. The reduced release of IL-1β could be associated with the reduced expression of NLRP3 seen during estradiol exposure of *P. gingivalis* or the decreased LDH release. Cell death, pyroptosis, has been shown to be directly linked to the release of IL-1β^37^. In addition, we have previously shown the proteolytic effect of gingipains on cytokines like IL-8 and IL-1β. At lower concentrations, arginine gingipain was very efficient at inducing proteolytic cleavage of the cytokines whereas at higher levels both arginine and lysine gingipains were equally potent at cleaving IL-8 and IL-1β^41^. The increased gingipain release associated with estradiol exposure might hence explain the lower IL-1β, CXCL10 and TGF-β1 levels.

We continued to evaluate the gene expression of pro-IL-1α, pro-IL-1β, IL-18, IL-6, IL-8 and TGF-β1, all previously associated with periodontitis^42,43^. We found that that the gene expression of IL-18, IL-6, IL-8 and TGF-β1 in gingival epithelial cells was significantly lower in *P. gingivalis* stimulated with 1 pg/ml estradiol. However, this difference was not observed for pro-IL-1α and pro-IL-1β. IL-1 family is comprised of several members, of which IL-1α, IL-1β and IL-18 are pro-inflammatory cytokines^44^. IL-6 is a transient cytokine secreted in response to infection and injury. Like IL-1β, tight regulation of IL-6 occurs at various levels of transcription and release, as dysregulation in this mechanism can lead to chronic inflammation such as periodontitis^42^. IL-8 is a chemokine strongly associated to the recruitment of neutrophils to the infection site and hence important during the infection^2,43^. We have previously shown that gingipains are involved in suppressing cytokine gene expression^24,45^, and the increased gingipains release we observed might partly explain the reduced gene expressions. In addition, gingipains have been shown to be very efficient at inducing proteolytic cleavage of cytokines which might explain deviations between gene and protein level^2^. However, further investigation is needed to evaluate why no correlation exists between the gene and protein level of IL-1β and TGF-β1.

Understanding how the human host affects the virulence of *P. gingivalis* may be a new frontier in the fight against *P. gingivalis* associated infections. If we successfully unravel how *P. gingivalis* senses its microenvironment and activates its virulence, we may be able to prevent or dampen this activation, which in turn could prevent the infection progression. We have seen that both pre- and postmenopausal concentration of estradiol could alter key virulence traits of *P. gingivalis*. Although we have shown that estradiol can alter the virulence of *P. gingivalis*, further investigation is needed to elucidate the mechanism behind these findings and what clinical significance they may have.

## Data Availability

All data generated or analysed during this study are included in this published article.
